# Epithelial Neuroendocrine Neoplasms: Grading Based on the New WHO 2022 Guidelines

**DOI:** 10.7759/cureus.102857

**Published:** 2026-02-02

**Authors:** Rizwana Shaik, Inuganti Venkata Renuka, Bakkamanthala S K Kanth, Leela Lahari Arikathota

**Affiliations:** 1 Pathology, NRI Medical College, Vijayawada, IND

**Keywords:** gep- gastroentero pancreatic, ihc – immunohistochemistry, mct – medullary carcinoma of thyroid, nec–neuroendocrine cracinoma, nen- neuroendocrine neoplasm, net- neuroendocrine tumour, pnet- pulmonary neuroendocrine tumour

## Abstract

Neuroendocrine tumors (NETs) are rare. Epithelial NETs are generally considered slow-growing tumors. Arising from neuroendocrine cells, NETs can develop at various primary sites in the body.

Gastrointestinal NETs are the most common of all NETs. The ileum, appendix, and rectum are among the most common sites, followed by the colon, stomach, and duodenum; some series report a female preponderance.

Bronchopulmonary NETs are relatively rare and are often indolent in nature.

In recent decades, the incidence has been rising. This may be due to greater awareness, improved diagnostic tools, or changes in definitions/classification. Grading of epithelial neuroendocrine neoplasms is beneficial, as it allows tumors to be graded by proliferative activity and distinguished from neuroendocrine carcinomas.

## Introduction

Neuroendocrine tumors (NETs) account for only 0.5% of all malignancies [1]. These are rare, with an annual incidence of 1 to 2 per 100,000, with a female preponderance of around 2.5:1 under the age of 50 years due to appendiceal location [2,3]. The age at presentation is related to the primary site. Appendiceal localization is most frequently seen at a young age, particularly in women [1]. They represent 2% of all tumors of the GI tract.

The primary sites are the GI tract (62% to 67%), especially the appendix (27%), followed by the small bowel (15%) and the lung (22% to 27%) [3]. Presentation with metastatic disease accounts for 12% to 22% [3]. Smoking or alcohol consumption does not appear to increase the risk of neuroendocrine neoplasms (NENs) [1].

Bronchopulmonary NETs are relatively rare and often indolent in nature, and account for 2% of primary lung cancers [4]. Small-cell lung cancer (SCLC), accounting for ~15% of all lung cancers, has become a leading cause of cancer-related mortality and morbidity worldwide [5].

NETs are generally known as slowly growing tumors, which may develop at any site and produce various peptides, especially serotonin. Neuroendocrine carcinomas (NECs) represent only 10% to 20% of all NENs [6].

To standardize the nomenclature of these NETs and resolve complexity, the WHO proposed a universal definition system for neuroendocrine neoplasia based on differentiation and proliferative grading [7].

NENs of non-endocrine organs were incorporated for the first time in the 2022 WHO Classification of Endocrine Tumors [7]. A major goal of the 2022 update was to unify the classification of NENs across all parts of the body, including the digestive, endocrine, head, and neck systems. The principles of grading are now consistently applied across different organs, with most NENs categorized as NETs (G1, G2, or G3) or NECs.

The aim of the study is to grade the epithelial NENs by the new WHO 2022 classification and investigate their association with various clinicopathological parameters. The objective is to grade all NETs by differentiation and proliferative activity under the unified new WHO 2022 classification.

Tumor grading is based on mitotic count and/or Ki67 (MIB-1 clone) labeling indices. Tumor necrosis, though recognized as a potential adverse prognostic factor, is not included in the grading parameters of tumors in the gastrointestinal and pancreaticobiliary tract; however, it is a component of tumor grading in other locations, such as the lung and thymus, as well as the upper aerodigestive tract [7].

Grading of gastroenteropancreatic (GEP) NETs is based on mitotic activity along with Ki67, which are key parameters used for grading (Grades 1, 2, and 3) and for distinguishing NEC. Lung and thymic NENs are classified as G1 NETs and G2 NETs, NETs with elevated mitotic counts and/or Ki67, and NEC.

The 2022 WHO Classification also introduces a 2-tiered grading system for medullary thyroid carcinomas based on mitotic count, Ki67 proliferative index, and tumor necrosis as low grade and high grade [7]. Pituitary adenomas have no formal grading system available [7]. All NETs express INSM1, synaptophysin, and chromogranin. Synaptophysin is considered the most sensitive neuroendocrine marker, whereas chromogranin A is the most specific.

## Materials and methods

The study was conducted in the Department of Pathology from 2022 to 2025 for a period of 3 years for histopathological evaluation and immunohistochemical (IHC) correlation, with ethical clearance (IEC 2026 F001). This is an observational, hospital-based study with 50 subjects who were diagnosed with NETs and NECs on endoscopic biopsies or resection specimens. Biopsy-proven cases were subjected to IHC staining for chromogranin, synaptophysin, and the Ki67 labeling index (Dako, Glostrup, Denmark).

The procedure begins with deparaffinization and rehydration of formalin-fixed, paraffin-embedded tissue sections, which are typically 4 microns in thickness. Antigen retrieval is then performed to unmask epitopes, followed by endogenous peroxidase blocking for 5 minutes. The tissue is then incubated for 30 minutes with a primary antibody specific for chromogranin or synaptophysin. Ki67 detection is achieved using the application of horseradish peroxidase for 30 minutes and chromogenic substrate for 10 minutes. Finally, the slides were counterstained with hematoxylin.

The inclusion criteria for this study are that all cases of epithelial NENs received at NRI Medical College are included. Inadequate samples are excluded. Each case of NET diagnosed on histopathology sections is included, and immunohistochemistry is performed for chromogranin, synaptophysin, and the Ki67 index. Cytoplasmic staining was positive for chromogranin and synaptophysin, and nuclear staining was positive for Ki67.

For precise evaluation of grading, a minimum of 50 high-power fields (HPFs) is reported as the sum of the ten highest counts for the mitotic count, and at least 500 cells are counted for the Ki67 labeling index from hotspots. Grading of the NETs was done according to the new WHO 2022 classification (Table 1).

**Table 1:** WHO 2022 grading of epithelial neuroendocrine neoplasms at different anatomical sites. NET: Neuroendocrine tumor; NEC: Neuroendocrine carcinoma; Ki-67: Ki-67 proliferation index; MTC: Medullary thyroid carcinoma; mm²: Square millimeters.

Anatomical site / system	Neuroendocrine neoplasm type	Classification	Diagnostic criteria
GI and pancreatobiliary tract	Well-differentiated NET	NET, grade 1	< 2 mitoses/2 mm² and/or Ki-67 < 3%
GI and pancreatobiliary tract	Well-differentiated NET	NET, grade 2	2-20 mitoses/2 mm² and/or Ki-67 < 20%
GI and pancreatobiliary tract	Well-differentiated NET	NET, grade 3	> 20 mitoses/2 mm² and/or Ki-67 > 20%; no necrosis
GI and pancreatobiliary tract	Poorly differentiated NEC	Small cell NEC	> 20 mitoses/2 mm² and/or Ki-67 > 20% (often > 70%), and small-cell cytomorphology
GI and pancreatobiliary tract	Poorly differentiated NEC	Large cell NEC	> 20 mitoses/2 mm² and/or Ki-67 > 20% (often > 70%), and large-cell cytomorphology
Upper aerodigestive tract and salivary glands	Well-differentiated NET	NET, grade 1	< 2 mitoses/2 mm², no necrosis, and Ki-67 < 20%
Upper aerodigestive tract and salivary glands	Well-differentiated NET	NET, grade 2	2-10 mitoses/2 mm² and/or necrosis, and Ki-67 < 20%
Upper aerodigestive tract and salivary glands	Well-differentiated NET	NET, grade 3	> 10 mitoses/2 mm² and/or Ki-67 > 20%
Upper aerodigestive tract and salivary glands	Poorly differentiated NEC	Small cell NEC	> 10 mitoses/2 mm² and/or Ki-67 > 20% (often > 70%), and small-cell cytomorphology
Upper aerodigestive tract and salivary glands	Poorly differentiated NEC	Large cell NEC	> 10 mitoses/2 mm² and/or Ki-67 > 20% (often > 55%), and large-cell cytomorphology
Lung and thymus	Well-differentiated NET	NET, grade 1	< 2 mitoses/2 mm² and no necrosis
Lung and thymus	Well-differentiated NET	NET, grade 2	2-10 mitoses/2 mm² and/or necrosis (usually punctate)
Lung and thymus	Carcinoids/NETs with elevated mitotic counts and/or Ki-67	Elevated counts	Atypical carcinoid morphology with > 10 mitoses/2 mm² and/or Ki-67 > 30%
Lung and thymus	Poorly differentiated NEC	Small cell carcinoma	> 10 mitoses/2 mm², often necrosis, and small-cell cytomorphology
Lung and thymus	Poorly differentiated NEC	Large cell NEC	> 10 mitoses/2 mm², virtually always necrosis, and large-cell cytomorphology
Thyroid	MTC	Low-grade MTC	< 5 mitoses/2 mm², no necrosis, and Ki-67 < 5%
Thyroid	MTC	High-grade MTC	At least one of: ≥ 5 mitoses/2 mm², necrosis, Ki-67 ≥ 5%

Statistical analysis

Quantitative variables were reported as mean and range and were compared using the Student’s t-test. Categorical variables were presented as numbers with frequencies as proportions (%) and were compared using the Chi-square test or Fisher’s exact test, as appropriate. A two-sided P value < 0.05 was considered statistically significant. Comparisons between groups were also performed using Fisher’s exact test and the Mann-Whitney U test, as appropriate. Tumor differentiation status, defined as Grade 1, 2, and 3 in GEP NENs, was analyzed in relation to prognosis. All statistical analyses were carried out using IBM SPSS Statistics version 25.0.

## Results

A total of 50 cases of epithelial NENs were studied. The age range was 46-82 years. The highest number of cases were in the age group of 50-58 years. There were males (20) and females (30) in this study, with a male-to-female ratio of 2:3 (Table 2).

**Table 2:** Characteristics of study population according to tumor differentiation status. NETs: Neuroendocrine tumors; NET: Neuroendocrine tumor; NEC: Neuroendocrine carcinoma; G1: Grade 1; G2: Grade 2; G3: Grade 3; N: Number; %: Percent; P: Probability (p-value); M: Male; F: Female; GEP: Gastroenteropancreatic; ca.: Carcinoma; NA: Not applicable.

Characteristics	Total no. of patients, N (%)	Well-differentiated NETs (G1, G2), N (%)	Poorly differentiated NETs (G3, NEC), N (%)	p-value
Number of cases	50 (100%)	39 (78%)	11 (22%)	
Biopsy	14 (28%)	9	5	0.048
Surgical resection	36 (72%)	30	6	0.001
Age (range; mean)	46-82 (64)	46-58 (52)	63-82 (72.5)	0.001
Sex (M, F)	20, 30	8, 9	12, 21	0.005
Male:female ratio	2:3	8:9	4:7	
Primary site: GEP	20 (40%)	16 (32%)	4 (8%)	0.371
- Stomach	4	4	0	
- Duodenum and pancreas	4	3	1	
- Small intestine	8	6	2	
- Rectum	4	3	1	
Primary site: lung	14 (28%)	12 (24%)	2 (4%)	0.285
Primary site: head and neck	1 (2%)	1	0	NA
Primary site: thyroid (medullary ca.)	5 (10%)	5	0	NA
Primary site: pituitary NET	8 (16%)	8	0	NA
Lymph nodes	1	1	0	NA
Perineural invasion	1	1	0	NA

Regarding the primary tumor site, tumors most commonly arose in the GEP region, with 20 cases (40%), followed by the lung with 14 cases (28%), one case (2%) in the head and neck region, eight cases (16%) of pituitary neuroendocrine tumor, and five cases (10%) of medullary carcinoma of thyroid (MCT). In GEP NENs, the distribution along the GI tract was stomach with 4 cases (20%), duodenum and pancreas with 4 cases (20%), small intestine with 8 cases (40%), and rectum with 4 cases (20%) (Table 3). In our study, the GEP tract was the most common primary site, in which mean mitotic activity and mean Ki67 indices of GEP-NENs are listed. For NENs at GEP location, the Chi-square test showed a χ² value of 0.8 and a p-value of 0.371, which was not significant. It is apparent that although poorly differentiated neuroendocrine carcinomas (PD-NECs) generally have a higher average proliferative index than well-differentiated neuroendocrine tumors (WD-NETs), review of the cases showed that mitotic activity appears to have influenced the classification of G3 WD-NETs (Table 3).

**Table 3:** Location of differentiation grades in GEP-NENs with mean mitotic and Ki67 indices. NEC: Neuroendocrine carcinoma; MA: Mitotic activity; Ki67: Ki-67 proliferation index; %: Percent.

Location (n)	Grade 1	Grade 2	Grade 3	NEC	Mean MA	Mean Ki67 (%)
Stomach (4)	3	1	0	0	1.16	1.6
Duodenum & Pancreas (4)	2	1	0	1	8.4	8.6
Small intestine (8)	5	1	0	2	10	10
Rectum (4)	2	1	1	0	9.4	12
Total (20)	12 (60%)	4 (20%)	1 (5%)	3 (15%)		

Out of 50 cases, there were 39 cases (78%) of neuroendocrine tumors and 11 cases (22%) of neuroendocrine carcinomas. Poorly differentiated tumors occurred in significantly older patients, which was highly significant (p < 0.001). A 20-year age difference was observed, suggesting age could be a prognostic factor. Females were more likely to have poorly differentiated tumors, which was significant (p < 0.05). Poorly differentiated tumors were more commonly diagnosed on biopsy specimens than in surgically resected cases. NECs occurred in older patients than NET G3 and were statistically significant (Table 4).

**Table 4:** Clinicopathological characteristics comparison of NET Grade 3 and NEC. NET: Neuroendocrine tumor; G3: Grade 3; NEC: Neuroendocrine carcinoma; n: Number (sample size); M: Male; F: Female; GEP: Gastroenteropancreatic.

Characteristic	NET G3 (n = 6)	NEC (n = 5)	P-value
Age (years)	46-58	63-82	0.016
Sex (M:F)	8:9	4:7	
GEP	1	3	0.242
Lung	5	2	0.239

Non-significant factors were primary site location within the GEP-NET group (p = 0.371) and overall site distribution (GEP vs lung) (p = 0.285). A strong correlation between mitotic activity and Ki-67 supports the current WHO 2022 grading system. By Fisher’s exact test, there appeared to be a gender association with differentiation status (p < 0.01). The majority of NETs in this study were NET G1 (25 cases; 50%), followed by NET G2 and NET G3 (six cases each; 12%). The Mann-Whitney U test showed that NECs occurred in significantly older patients than NET G3 tumors, suggesting age-related progression to more aggressive histology (P = 0.016). Fisher’s exact test was also performed and showed no statistical significance, with a two-tailed P-value of 0.242, an odds ratio of 0.267, and a 95% CI of 0.018-3.95. Based on this comparison, NECs were evenly distributed, with a slight GEP preference (60%). The trend suggests that lung NETs are more likely to fall under neuroendocrine tumors with high mitotic activity, and GEP-NETs are more likely to be NECs (Table 4).

**Figure 1:**
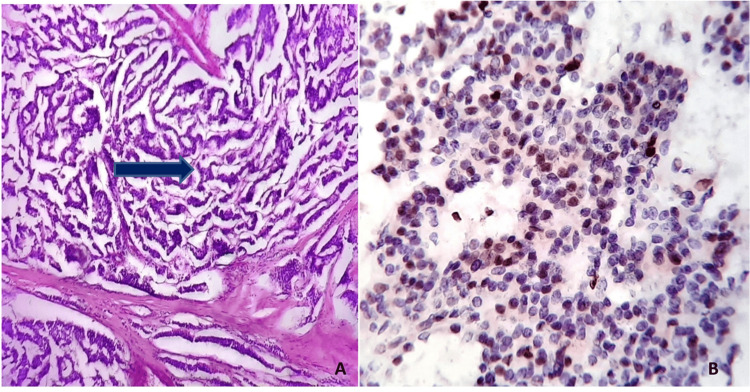
Well-differentiated neuroendocrine tumor. A) H&E stain, 400×: The blue arrow indicates a well-differentiated neuroendocrine tumor with a trabecular pattern and a mitotic activity of 2-20/10 HPF. B) IHC (Ki67), 400×: Shows < 20% positivity (Grade 1). IHC: Immunohistochemistry; Ki67: Ki-67 proliferation index; HPF: High-power field; ×: Magnification (times).

All WD-NET cases revealed classic histopathologic features of WD-NET, which included an organoid, trabecular, and insular architecture; a regular intratumoral vascular pattern; abundant granular cytoplasm; and stippled nuclei with inconspicuous nucleoli (Figure 1).

**Figure 2:**
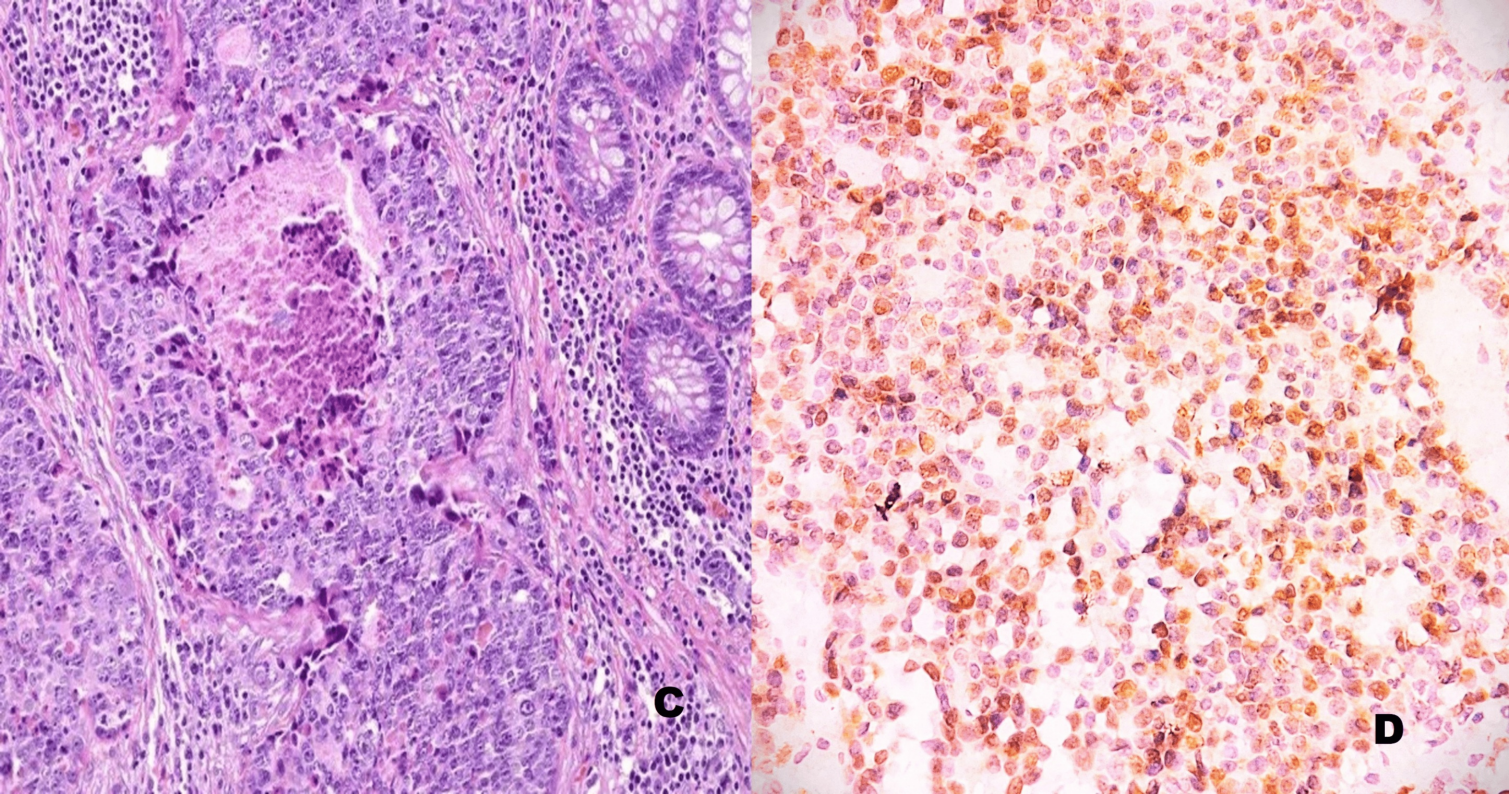
Poorly differentiated neuroendocrine tumor of the small intestine. C) H&E stain, 400×: Shows a neuroendocrine tumor with mitotic activity of > 20 per 10 HPF (Grade 3). D) Immunohistochemistry (Ki-67), 400×: Shows > 20% positivity. IHC: Immunohistochemistry; Ki67: Ki-67 proliferation index; HPF: High-power field; ×: Magnification (times)

Five cases of PD-NEC (small cell carcinoma) showed geographic tumor necrosis, minimal cytoplasm, finely granular chromatin, hyperchromatic nuclei with inconspicuous nucleoli, and nuclear molding (Figure 2).

**Figure 3:**
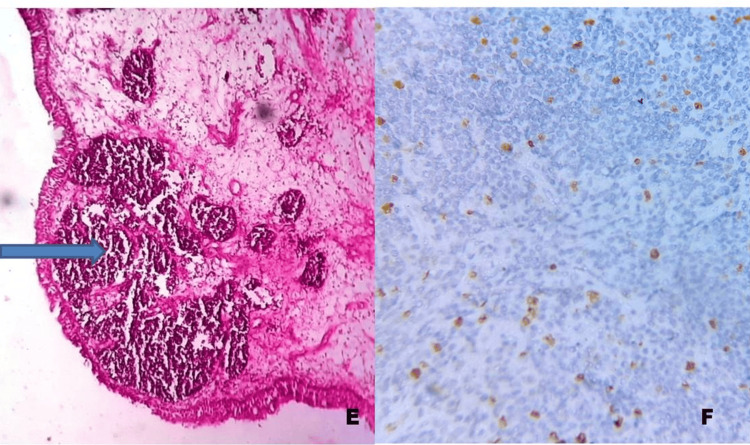
Pulmonary neuroendocrine tumor. E) H&E stain, 400×: shows a neuroendocrine tumor in the bronchus. F) Immunohistochemistry (Ki-67), 400×: Shows < 10% positivity (Grade 1). Ki67: Ki-67 proliferation index; ×: Magnification (times).

**Figure 4:**
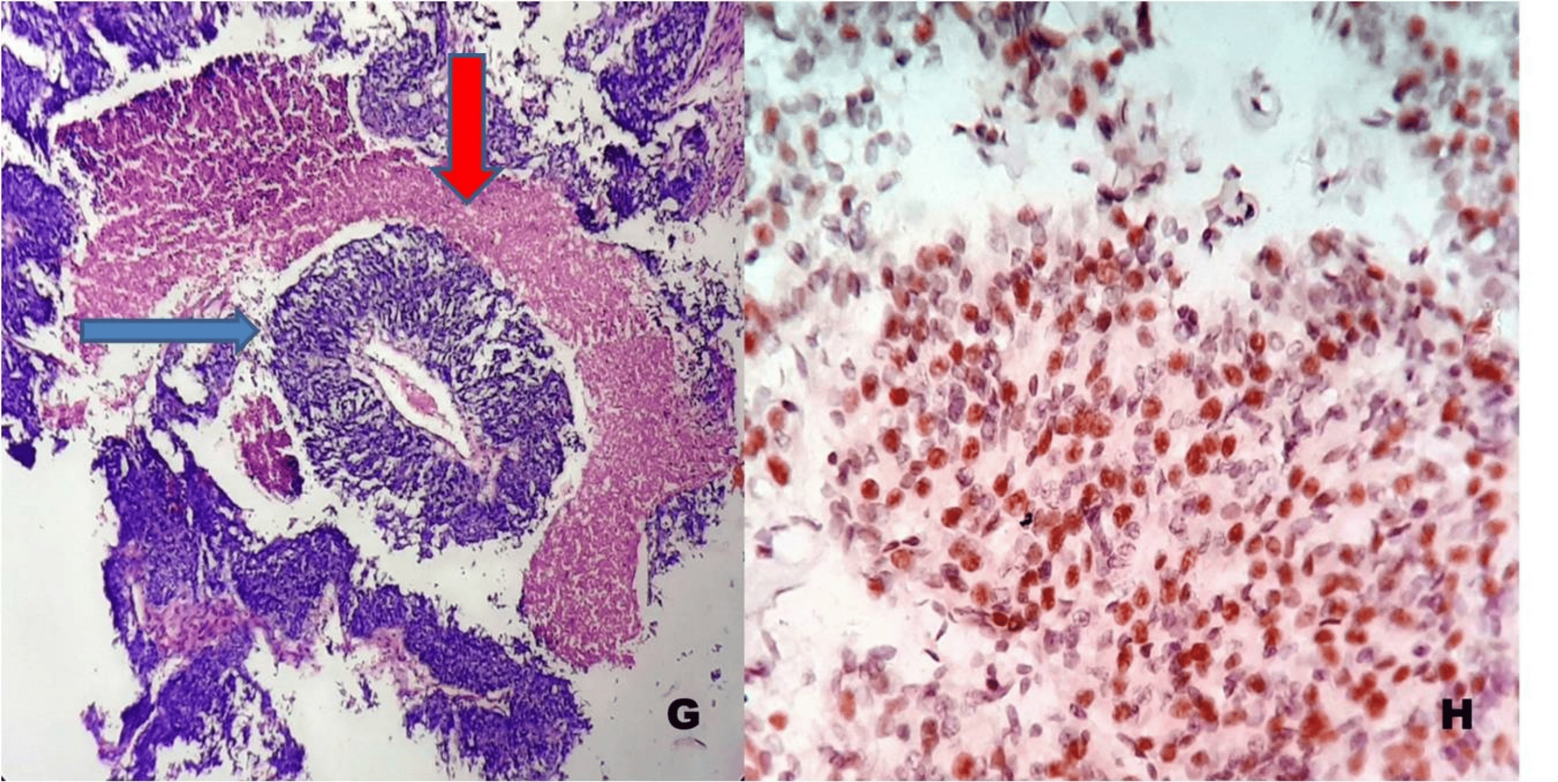
Poorly differentiated pulmonary neuroendocrine tumor. G) H&E stain, 400×: The blue arrow indicates tumor cells, and the red arrow indicates tumor necrosis in small cell carcinoma of the lung. H) Immunohistochemistry (Ki-67), 400×: The blue arrow indicates > 20% positivity (Grade 3). Ki67: Ki-67 proliferation index; ×: Magnification (times).

Lung NETs were identified in 14 cases: five cases (35.7%) were Grade 1, six cases (42.8%) were Grade 2, and three cases (21.4%) were NEC by the previous WHO classification (Figures 3-4).

Out of the six Grade 2 cases previously classified under the previous WHO classification, four cases were regrouped (upgraded) into carcinoids/NETs with elevated mitotic counts and/or Ki67 proliferation index, as the mitotic count crossed the limit of 10 mitoses/10 HPF and the Ki67 index was 30% (Table 5).

**Table 5:** Comparison and regrading of lung NENs by the old (2017) and new (2022) WHO classifications. NET: Neuroendocrine tumor; NENs: Neuroendocrine neoplasms; NEC: Neuroendocrine carcinoma; n: Number (sample size); %: Percent; Ki67: Ki-67 proliferation index.

Lung NET by older WHO	No. of cases, n (%)	Lung NENs (WHO 2022)	No. of cases, n (%)
Grade 1	5 (35.7%)	Grade 1	5 (35.7%)
Grade 2	6 (42.8%)	Grade 2	2 (14.3%)
NEC	3 (21.4%)	Carcinoids/NETs with elevated mitotic count and/or Ki67 proliferation index	5 (35.7%)
		NEC	2 (14.2%)

**Figure 5:**
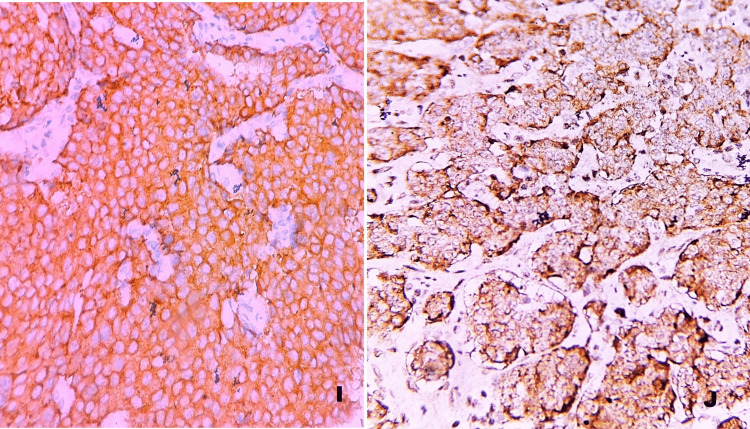
Synaptophysin and chromogranin positivity. I) IHC, 400×: Chromogranin - cytoplasmic positivity. J) IHC, 400×: Synaptophysin - cytoplasmic positivity.

In the poorly differentiated group, one case previously classified as neuroendocrine carcinoma was downgraded to carcinoids/NETs with elevated mitotic counts and/or Ki67 proliferation index, as the Ki67 index was only 30%. All cases were positive for chromogranin and synaptophysin (Figure 5).

**Figure 6:**
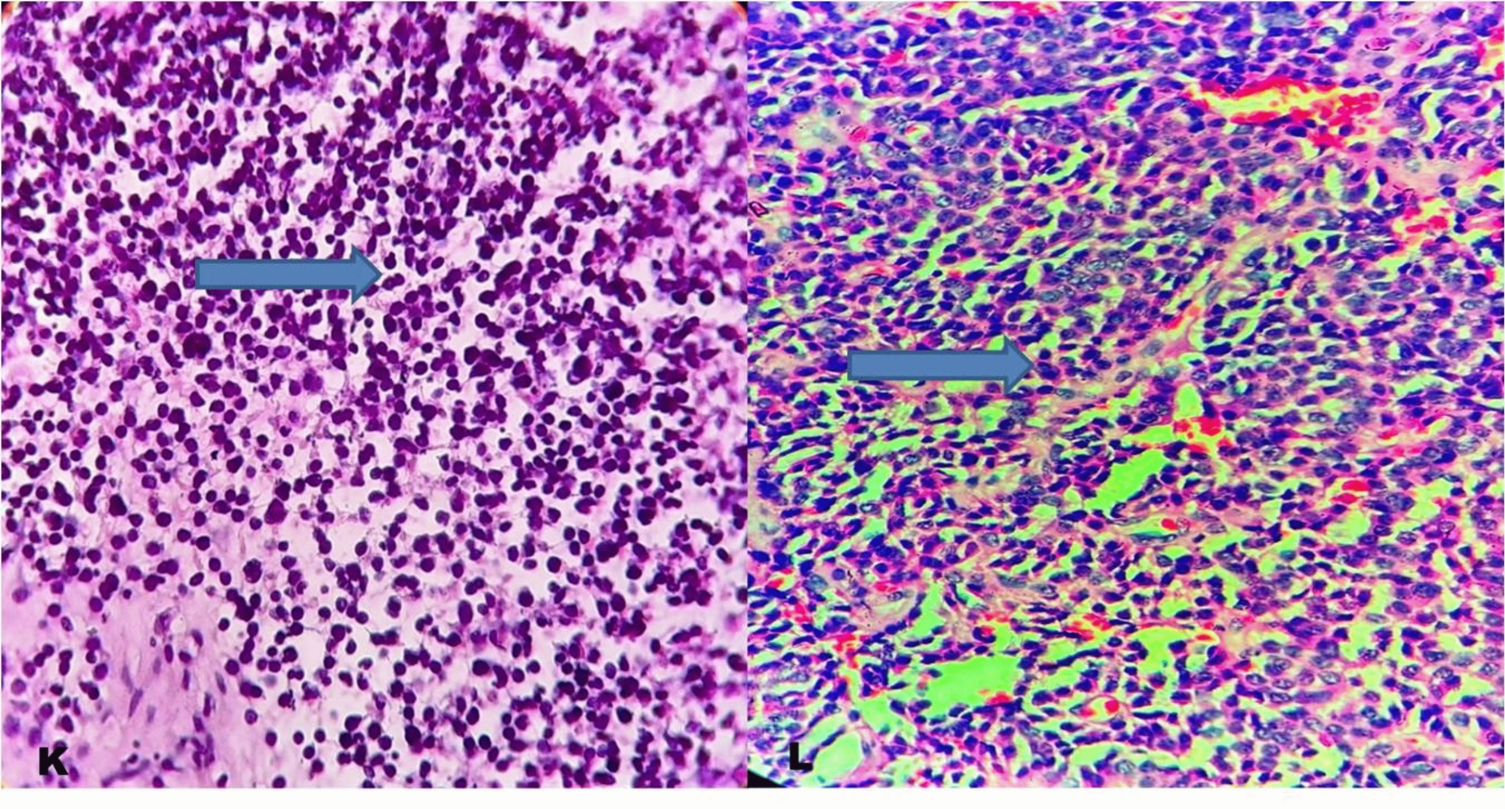
Pituitary neuroendocrine tumor. (K and L) H&E stain, 400×: The blue arrow indicates a pituitary neuroendocrine tumor.

**Figure 7:**
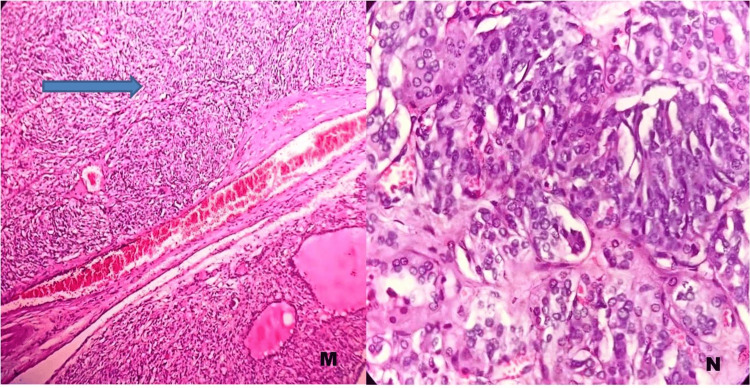
Medullary carcinoma of the thyroid. M) H&E stain, 100×: Medullary carcinoma of the thyroid. N) H&E stain, 400×: Medullary carcinoma of the thyroid with low mitotic activity (low grade).

All eight (16%) cases of pituitary neuroendocrine tumor and five (10%) cases of medullary carcinoma of the thyroid were of lower grade, with no necrosis or mitotic activity noted (Figures 6-7).

## Discussion

Derived from neural crest cells during development, NETs can arise from various primary sites in the body. NENs arising from the bronchopulmonary and GEP systems account for over 90% of all NENs [8]. In the lung, well-differentiated tumors are more prevalent, accounting for 27%, with Grade 1 tumors more prevalent than Grade 2 NETs, accounting for 90% of the well-differentiated category [9]. Regardless of stage and primary location, poorly differentiated GEP-NENs have a poorer outcome than well-differentiated GEP-NENs.

GEP-NENs are a complex family of neoplasms with widely variable biological behavior. Multiple factors, such as proliferation rate, histological differentiation, and tumor site, are important determinants of tumor stage. The distribution patterns of NETs in the GI tract seem to differ between Eastern and Western populations [1]. The most common location of NETs in the GI tract among patients in the United States is the small intestine, followed by the pancreas, according to the analysis by Dasari A et al. [10], which agrees with our study, as the GI tract is the most common site, followed by the small intestine. Two other studies from Denmark and Sweden also report small intestine NETs as most prevalent [11,12].

The rectum (48%) is the most frequent location of NETs in the GI tract among patients in Korea, followed by the stomach (15%), pancreas (9%), colon (8%), small intestine (8%), liver (7%), appendix (3%), and biliary tract (2%) [1]. Incidences of NETs in other parts of the GI tract were unchanged in one study of the Korean population [13]. Appendiceal NETs were prevalent in Western countries, whereas rectal NETs were common in Asian populations [14,15]. In our study, across all age groups, GEP NET incidence rose linearly. The >50 age group demonstrated a higher absolute incidence rate compared to patients <50 years. The maximum incidence rate was observed in patients >60 years. When examining incidence by primary site of disease, patients with small intestinal, colonic, rectal, appendiceal, and pancreatic NETs all experienced increasing incidence rates across age groups.

Comparison with nine other studies of GEP NENs, Vélayoudom-Céphise FL et al. [16], Abdulfattah MK and Al-naqqash MA [17], Tang LH et al. [18], Xue Y et al. [19], Tang LH et al. [20], Yang M et al. [21], Prakash PS and Wijerathne S [22], Uppin MS et al. [23], and Darbà J and Marsà A [24], shows that our findings agree with Uppin MS et al. [23], in which the small intestine was the most common site, whereas most other studies report the pancreas as the most common site of NETs. In our study, the age range was 46-82 years, with a higher number of female patients, which is consistent with Tang LH et al. and Yang M et al. [18,21]. Other studies, such as Vélayoudom-Céphise FL et al. [16], Yang M et al. [21], and Darbà J and Marsà A [24], reported Grade 3 as the most frequent grade; however, in our study there was only one case of Grade 3 GEP-NEN.

Among the 11 WD-NENs, four tumors showed a Ki67 index > 20% (WD-NEN Ki67 > 20%), which accounted for 28.4% of all WD-NENs and 28.5% of all lung NENs. All WD-NENs with Ki67 > 20% were found among Grade 1 and Grade 2 tumors. This stratification identified four WD-NENs with a Ki67 index > 20%, falling into the carcinoids/NETs with elevated mitotic count and/or Ki67 proliferation index category. 

Across studies, NETs generally fall into a middle-age group of around 40-80 years, and Kasajima A et al. (2018) [25] reported a predominant number of cases with high mitotic activity. In our study, there was a female preponderance in lung NETs, which is consistent with the study by Kasajima A and Klöppel G (2022) [8], while other studies such as Quinn AM et al. [26] and Oka N et al. [27] show a male preponderance. Tumors that shifted into the carcinoids/NETs with elevated mitotic count and/or Ki67 proliferation index category originally belonged to the poorly differentiated tumors; however, despite having a mitotic count of 30 MA/2 mm², they showed rather well-differentiated morphology.

## Conclusions

The increasing incidence of NETs has been reported, accompanied by increased awareness and improved endoscopic methods of detection at our hospital. In our study, the number of NET G3 cases is limited. Lung NET G3 is comparable to NET G3 in other organs; grading is conceivable and should be realized in a future WHO classification.

The current WHO 2022 classification represents an important step forward in facilitating the diagnosis of NENs and, with continued understanding of this unique group of neoplasms, may impact prognosis.
